# Risk for Stillbirth Among Women With and Without COVID-19 at Delivery Hospitalization — United States, March 2020–September 2021

**DOI:** 10.15585/mmwr.mm7047e1

**Published:** 2021-11-26

**Authors:** Carla L. DeSisto, Bailey Wallace, Regina M. Simeone, Kara Polen, Jean Y. Ko, Dana Meaney-Delman, Sascha R. Ellington

**Affiliations:** 1 CDC COVID-19 Response Team.

Pregnant women are at increased risk for severe COVID-19–related illness, and COVID-19 is associated with an increased risk for adverse pregnancy outcomes and maternal and neonatal complications (*1*–*3*). To date, studies assessing whether COVID-19 during pregnancy is associated with increased risk for stillbirth have yielded mixed results (*2*–*4*). Since the B.1.617.2 (Delta) variant of SARS-CoV-2 (the virus that causes COVID-19) became the predominant circulating variant,* there have been anecdotal reports of increasing rates of stillbirths in women with COVID-19.^†^ CDC used the Premier Healthcare Database Special COVID-19 Release (PHD-SR), a large hospital-based administrative database,^§^ to assess whether a maternal COVID-19 diagnosis documented at delivery hospitalization was associated with stillbirth during March 2020*–*September 2021 as well as before and during the period of Delta variant predominance in the United States (March 2020*–*June 2021 and July–September 2021, respectively). Among 1,249,634 deliveries during March 2020–September 2021, stillbirths were rare (8,154; 0.65%): 273 (1.26%) occurred among 21,653 deliveries to women with COVID-19 documented at the delivery hospitalization, and 7,881 (0.64%) occurred among 1,227,981 deliveries without COVID-19. The adjusted risk for stillbirth was higher in deliveries with COVID-19 compared with deliveries without COVID-19 during March 2020–September 2021 (adjusted relative risk [aRR] = 1.90; 95% CI = 1.69–2.15), including during the pre-Delta (aRR = 1.47; 95% CI = 1.27–1.71) and Delta periods (aRR = 4.04; 95% CI = 3.28–4.97). COVID-19 documented at delivery was associated with increased risk for stillbirth, with a stronger association during the period of Delta variant predominance. Implementing evidence-based COVID-19 prevention strategies, including vaccination before or during pregnancy, is critical to reducing the impact of COVID-19 on stillbirths.

Delivery hospitalizations were identified from PHD-SR using *International Classification of Diseases, Tenth Revision, Clinical Modification* (ICD-10-CM) diagnostic and procedure codes pertaining to obstetric delivery and diagnosis-related group delivery codes.^¶^ Deliveries with discharge dates during March 2020–September 2021 were included. Stillbirths, defined as fetal deaths at ≥20 weeks’ gestation, were identified using maternal ICD-10-CM diagnosis codes.** Hospitalizations without ICD-10-CM codes indicating gestational age or with ICD-10-CM codes indicating gestational age <20 weeks were excluded to reduce misclassification of fetal deaths at <20 weeks’ gestation as stillbirths (1.5% of the overall sample).

Maternal demographic variables assessed included age, race/ethnicity (i.e., Hispanic, non-Hispanic Black, non-Hispanic White, non-Hispanic Asian, and non-Hispanic other), and primary payor (i.e., Medicaid, private insurance, self-pay, and other). Assessed hospital characteristics included urban or rural location and U.S. Census division. COVID-19^††^ and selected underlying medical conditions (i.e., obesity, smoking,^§§^ any diabetes,^¶¶^ any hypertension,*** and multiple-gestation pregnancy) were included if the relevant ICD-10-CM diagnosis code was documented during the delivery hospitalization (*3*). In addition, among deliveries with documented COVID-19, indicators of severe illness (i.e., adverse cardiac event/outcome,^†††^ placental abruption, sepsis, shock, acute respiratory distress syndrome, mechanical ventilation, and intensive care unit [ICU] admission) were considered present if the relevant ICD-10-CM diagnosis code was documented during the delivery hospitalization (*3*). Vaccination status was unable to be assessed in this analysis.

Poisson regression models with robust standard errors were used to calculate overall unadjusted and adjusted^§§§^ relative risks for stillbirth among deliveries with COVID-19 versus deliveries without COVID-19, accounting for within-hospital and within-woman correlation. To better understand the potential biologic mechanism for stillbirth among women with COVID-19 at delivery, Poisson regression models with robust SEs were used to calculate unadjusted and adjusted^¶¶¶^ prevalence ratios for stillbirth for each underlying medical condition and indicator of severe illness among deliveries with documented COVID-19. Relative risks and prevalence ratios were calculated overall as well as during the pre-Delta and Delta periods. Effect modification by period was assessed using adjusted models with interaction terms. For all models, p-values <0.05 were considered statistically significant. All analyses were performed using SAS software (version 9.4; SAS Institute). This activity was reviewed by CDC and was conducted consistent with applicable federal law and CDC policy.****

Among 1,249,634 deliveries at 736 hospitals during March 2020**–**September 2021, 53.7% of women were non-Hispanic White, and 50.6% had private insurance as the primary payor (Table 1). Overall, 15.4% had obesity, 11.2% had diabetes, 17.2% had a hypertensive disorder, 1.8% had a multiple-gestation pregnancy, and 4.9% had smoking (tobacco) documented on the delivery hospitalization record. Overall, 21,653 (1.73%) delivery hospitalizations had COVID-19 documented.

**TABLE 1:** Maternal demographic and health characteristics and hospital characteristics among delivery hospitalizations with and without a documented COVID-19 diagnosis — Premier Healthcare Database Special COVID-19 Release, United States, March 2020–September 2021

Characteristic	No. (%)						
	Overall N = 1,249,634			Pre-Delta* (Mar 2020–Jun 2021) n = 1,076,745		Delta* (Jul–Sep 2021) n = 172,889	
	Total N = 1,249,634	No COVID-19 n = 1,227,981	COVID-19 n = 21,653	No COVID-19 n = 1,058,651	COVID-19 n = 18,094	No COVID-19 n = 169,330	COVID-19 n = 3,559
**Maternal age, median (SD)**	**29.0 (5.8)**	29.0 (5.8)	28.0 (6.0)	29.0 (5.8)	28.0 (6.0)	29.0 (5.7)	28.0 (5.8)
**Maternal race/ethnicity**							
White, non-Hispanic	**671,392 (53.7)**	663,136 (54.0)	8,256 (38.1)	574,368 (54.3)	6,660 (36.8)	88,768 (52.4)	1,596 (44.8)
Hispanic	**230,836 (18.5)**	223,784 (18.2)	7,052 (32.6)	188,114 (17.8)	6,164 (34.1)	35,670 (21.1)	888 (25.0)
Black, non-Hispanic	**181,143 (14.5)**	177,508 (14.5)	3,635 (16.8)	153,408 (14.5)	2,947 (16.3)	24,100 (14.2)	688 (19.3)
Asian	**57,535 (4.6)**	56,855 (4.6)	680 (3.1)	49,583 (4.7)	604 (3.3)	7,272 (4.3)	76 (2.1)
Other/Unknown, non-Hispanic	**108,728 (8.7)**	106,698 (8.7)	2,030 (9.4)	93,178 (8.8)	1,719 (9.5)	13,520 (8.0)	311 (8.7)
**Primary payor**							
Private	**631,894 (50.6)**	624,069 (50.8)	7,825 (36.1)	537,957 (50.8)	6,367 (35.2)	86,112 (50.9)	1,458 (41.0)
Medicaid	**534,139 (42.7)**	521,739 (42.5)	12,400 (57.3)	450,813 (42.6)	10,548 (58.3)	70,926 (41.9)	1,852 (52.0)
Self-pay	**21,022 (1.7)**	20,557 (1.7)	465 (2.1)	17,351 (1.6)	386 (2.1)	3,206 (1.9)	79 (2.2)
Other	**62,579 (5.0)**	61,616 (5.0)	963 (4.4)	52,530 (5.0)	793 (4.4)	9,086 (5.4)	170 (4.8)
**Hospital location**							
Rural	**159,634 (12.8)**	157,006 (12.8)	2,628 (12.1)	134,615 (12.7)	2,014 (11.1)	22,391 (13.2)	614 (17.3)
Urban	**1,090,000 (87.2)**	1,070,975 (87.2)	19,025 (87.9)	924,036 (87.3)	16,080 (88.9)	146,939 (86.8)	2,945 (82.7)
**U.S. Census division**							
East North Central	**200,701 (16.1)**	198,061 (16.1)	2,640 (12.2)	169,631 (16.0)	2,259 (12.5)	28,430 (16.8)	381 (10.7)
East South Central	**94,224 (7.5)**	92,902 (7.6)	1,322 (6.1)	80,335 (7.6)	1,018 (5.6)	12,567 (7.4)	304 (8.5)
Middle Atlantic	**147,774 (11.8)**	144,423 (11.8)	3,351 (15.5)	124,755 (11.8)	3,123 (17.3)	19,668 (11.6)	228 (6.4)
Mountain	**91,554 (7.3)**	90,458 (7.4)	1,096 (5.1)	77,393 (7.3)	939 (5.2)	13,065 (7.7)	157 (4.4)
New England	**25,158 (2.0)**	24,892 (2.0)	266 (1.2)	21,463 (2.0)	246 (1.4)	3,429 (2.0)	20 (0.6)
Pacific	**126,615 (10.1)**	124,277 (10.1)	2,338 (10.8)	107,760 (10.2)	1,890 (10.4)	16,517 (9.8)	448 (12.6)
South Atlantic	**332,317 (26.6)**	326,419 (26.6)	5,898 (27.2)	283,595 (26.8)	4,683 (25.9)	42,824 (25.3)	1,215 (34.1)
West North Central	**80,263 (6.4)**	78,710 (6.4)	1,553 (7.2)	66,326 (6.3)	1,310 (7.2)	12,384 (7.3)	243 (6.8)
West South Central	**151,028 (12.1)**	147,839 (12.0)	3,189 (14.7)	127,393 (12.0)	2,626 (14.5)	20,446 (12.1)	563 (15.8)
**Obesity**							
No	**1,057,646 (84.6)**	1,039,849 (84.7)	17,797 (82.2)	897,069 (84.7)	14,881 (82.2)	142,780 (84.3)	2,916 (81.9)
Yes	**191,988 (15.4)**	188,132 (15.3)	3,856 (17.8)	161,582 (15.3)	3,213 (17.8)	26,550 (15.7)	643 (18.1)
**Diabetes (any)^†^**							
No	**1,109,053 (88.8)**	1,090,087 (88.8)	18,966 (87.6)	940,575 (88.8)	15,803 (87.3)	149,512 (88.3)	3,163 (88.9)
Yes	**140,581 (11.2)**	137,894 (11.2)	2,687 (12.4)	118,076 (11.2)	2,291 (12.7)	19,818 (11.7)	396 (11.1)
**Hypertensive disorders of pregnancy (any)^§^**							
No	**1,034,519 (82.8)**	1,016,918 (82.8)	17,601 (81.3)	877,063 (82.8)	14,678 (81.1)	139,855 (82.6)	2,923 (82.1)
Yes	**215,115 (17.2)**	211,063 (17.2)	4,052 (18.7)	181,588 (17.2)	3,416 (18.9)	29,475 (17.4)	636 (17.9)
**Multiple-gestation pregnancy**							
No	**1,226,534 (98.2)**	1,205,299 (98.2)	21,235 (98.1)	1,039,095 (98.2)	17,751 (98.1)	166,204 (98.2)	3,484 (97.9)
Yes	**23,100 (1.8)**	22,682 (1.8)	418 (1.9)	19,556 (1.8)	343 (1.9)	3,126 (1.8)	75 (2.1)
**Smoking^¶^**							
No	**1,187,831 (95.1)**	1,166,855 (95.0)	20,976 (96.9)	1,005,234 (95.0)	17,598 (97.3)	161,621 (95.4)	3,378 (94.9)
Yes	**61,803 (4.9)**	61,126 (5.0)	677 (3.1)	53,417 (5.0)	496 (2.7)	7,709 (4.6)	181 (5.1)
**Stillbirth**							
No	**1,241,480 (99.3)**	1,220,100 (99.4)	21,380 (98.7)	1,051,845 (99.4)	17,917 (99.0)	168,255 (99.4)	3,463 (97.3)
Yes	**8,154 (0.7)**	7,881 (0.6)	273 (1.3)	6,806 (0.6)	177 (1.0)	1,075 (0.6)	96 (2.7)
Timing of stillbirth, wks (trimester)**							
20–27 (2nd)	**3,607 (44.2)**	3,498 (44.4)	109 (39.9)	3,058 (44.9)	77 (43.5)	440 (40.9)	32 (33.3)
28–42 (3rd)	**4,547 (55.8)**	4,383 (55.6)	164 (60.1)	3,748 (55.1)	100 (56.5)	635 (59.1)	64 (66.7)
**Gestational age at stillbirth, wks, median (SD)**	**29.0 (6.8)**	29.0 (6.8)	29.0 (6.2)	29.0 (6.8)	29.0 (6.5)	30.0 (6.7)	30.0 (5.7)

**Abbreviation:** HELLP = hemolysis, elevated liver enzymes, low platelet count.

* Deliveries with discharge dates during March 2020–June 2021 were considered to have occurred during the pre-Delta period, whereas deliveries with discharge dates during July–September 2021 were considered to have occurred during the period of Delta predominance.

^†^ Includes prepregnancy diabetes and gestational diabetes.

^§^ Includes chronic hypertension, gestational hypertension, chronic hypertension with superimposed preeclampsia, preeclampsia, HELLP syndrome, and eclampsia.

^¶^ Includes smoking (tobacco) complicating pregnancy, childbirth, or the puerperium.

** Only among deliveries with a stillbirth.

During March 2020–September 2021, a total of 8,154 stillbirths were documented, affecting 0.64% and 1.26% of deliveries without COVID-19 and with COVID-19, respectively (aRR = 1.90; 95% CI = 1.69–2.15) (Figure). During the pre-Delta period (March 2020–June 2021), 6,983 stillbirths were documented, involving 0.98% of deliveries with COVID-19 compared with 0.64% of deliveries without COVID-19 (aRR = 1.47; 95% CI = 1.27–1.71). During the Delta period (July–September 2021), 1,171 stillbirths were documented, involving 2.70% of deliveries with COVID-19 compared with 0.63% of deliveries without COVID-19 (aRR = 4.04; 95% CI = 3.28–4.97).^††††^ Effect modification was present in the model; the risk for stillbirth was significantly higher during the period of Delta predominance than during the pre-Delta period (p<0.001).

**FIGURE:**
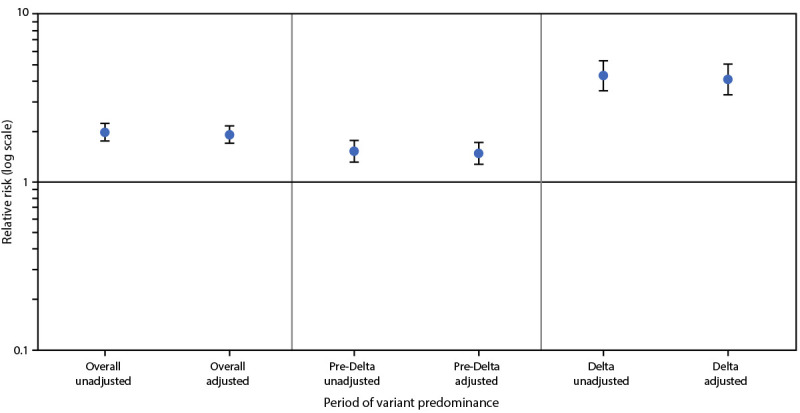
Relative risk for stillbirth among women with COVID-19 at delivery hospitalization compared with those without COVID-19 at delivery hospitalization — Premier Healthcare Database Special COVID-19 Release, United States, March 2020–September 2021*^,†,^^§^ **Abbreviation:** RR = relative risk. * Deliveries with discharge dates during March 2020–June 2021 were considered to have occurred during the period preceding SARS-CoV-2 B.1.617.2 (Delta) variant predominance, whereas those with discharge dates during July–September 2021 were considered to have occurred during the period of Delta predominance. ^†^ Overall: unadjusted RR = 1.96 (95% CI = 1.74–2.21); adjusted RR = 1.90 (95% CI = 1.69–2.15); pre-Delta: unadjusted RR = 1.52 (95% CI = 1.31–1.77); adjusted RR = 1.47 (95% CI = 1.27–1.71); Delta: unadjusted RR = 4.25 (95% CI = 3.46–5.22); adjusted RR = 4.04 (95% CI = 3.28–4.97); p-value for effect modification by period (pre-Delta period versus period of Delta predominance): <0.001. ^§^ Models accounted for within-facility and within-woman correlation, and were adjusted for maternal age, race/ethnicity (Hispanic, non-Hispanic Black, non-Hispanic White, and non-Hispanic other), primary payor (Medicaid, private insurance, and other), obesity, smoking, any diabetes, any hypertension, and multiple-gestation pregnancy.

Among deliveries with COVID-19, chronic hypertension, multiple-gestation pregnancy, adverse cardiac event/outcome, placental abruption, sepsis, shock, acute respiratory distress syndrome, mechanical ventilation, and ICU admission were associated with a higher prevalence of stillbirth (Table 2). The associations for adverse cardiac event/outcome and ICU admission varied significantly between the periods before and during Delta predominance (p = 0.03 and p = 0.003, respectively); for each of these, the associations were stronger during the period of Delta predominance.

**TABLE 2:** Risk for stillbirth by maternal health characteristics and indicators of severe illness among delivery hospitalizations with a documented COVID-19 diagnosis — Premier Healthcare Database Special COVID-19 Release, United States, March 2020–September 2021

Characteristic	Overall N = 21,653				Pre-Delta* (Mar 2020–Jun 2021) n = 18,094				Delta* (Jul–Sep 2021) n = 3,559				p-value^§^
	Outcome No. (%)		RR (95% CI)		Outcome No. (%)		RR (95% CI)		Outcome No. (%)		RR (95% CI)		
	No stillbirth	Stillbirth	Unadjusted	Adjusted^†^	No stillbirth	Stillbirth	Unadjusted	Adjusted^†^	No stillbirth	Stillbirth	Unadjusted	Adjusted^†^	
Hypertensive disorders of pregnancy (any)^¶^	3,995 (18.7)	57 (20.9)	1.15 (0.86–1.53)	1.08 (0.81–1.44)	3,379 (18.9)	37 (20.9)	1.14 (0.79–1.63)	1.05 (0.73–1.50)	616 (17.8)	20 (20.8)	1.21 (0.74–1.96)	1.19 (0.74–1.92)	<0.001
Chronic hypertension	515 (2.4)	13 (4.8)	2.00 (1.15–3.47)	1.79 (1.03–3.11)	418 (2.3)	7 (4.0)	1.71 (0.81–3.62)	1.49 (0.70–3.19)	97 (2.8)	6 (6.3)	2.24 (1.00–4.99)	2.11 (0.94–4.74)	0.02
Pregnancy-associated hypertension**	3,480 (16.3)	44 (16.1)	0.99 (0.72–1.36)	0.94 (0.68–1.29)	2,961 (16.5)	30 (16.9)	1.03 (0.70–1.52)	0.97 (0.66–1.43)	519 (15.0)	14 (14.6)	0.97 (0.66–1.43)	0.96 (0.55–1.69)	0.005
Obesity	3,810 (17.8)	46 (16.8)	0.94 (0.68–1.28)	0.90 (0.66–1.23)	3,181 (17.8)	32 (18.1)	1.02 (0.70–1.50)	0.97 (0.66–1.42)	629 (18.2)	14 (14.6)	0.77 (0.44–1.36)	0.78 (0.44–1.37)	0.02
Diabetes (any)^††^	2,659 (12.4)	28 (10.3)	0.81 (0.55–1.19)	0.80 (0.53–1.18)	2,273 (12.7)	18 (10.2)	0.78 (0.48–1.27)	0.78 (0.47–1.30)	386 (11.1)	10 (10.4)	0.93 (0.49–1.77)	0.88 (0.46–1.67)	0.005
Smoking^§§^	663 (3.1)	14 (5.1)	1.67 (0.98–2.85)	1.56 (0.91–2.68)	488 (2.7)	8 (4.5)	1.68 (0.83–3.39)	1.60 (0.79–3.27)	175 (5.1)	6 (6.3)	1.24 (0.55–2.80)	1.09 (0.47–2.52)	0.18
Multiple-gestation pregnancy	399 (1.9)	19 (7.0)	3.80 (2.41–6.00)	3.54 (2.24–5.59)	330 (1.8)	13 (7.3)	4.10 (2.36–7.14)	3.76 (2.16–6.57)	69 (2.0)	6 (6.3)	3.10 (1.40–6.85)	3.04 (1.35–6.82)	0.11
Adverse cardiac event/outcome^¶¶^	160 (0.7)	10 (3.7)	4.81 (2.60–8.87)	4.44 (2.38–8.29)	120 (0.7)	4 (2.3)	3.35 (1.26–8.89)	3.09 (1.15–8.34)	40 (1.2)	6 (6.3)	5.09 (2.35–11.03)	5.18 (2.34–11.48)	0.03
Placental abruption	273 (1.3)	36 (13.2)	10.49 (7.53–14.63)	10.12 (7.28–14.08)	206 (1.1)	22 (12.4)	11.12 (7.26–17.05)	10.63 (6.96–16.22)	67 (1.9)	14 (14.6)	7.33 (4.35–12.36)	7.53 (4.47–12.66)	0.07
Sepsis	306 (1.4)	10 (3.7)	2.57 (1.38–4.78)	2.55 (1.37–4.76)	211 (1.2)	6 (3.4)	2.89 (1.30–6.45)	2.83 (1.27–6.31)	95 (2.7)	4 (4.2)	1.52 (0.57–4.05)	1.58 (0.59–4.21)	0.56
Shock	121 (0.6)	15 (5.5)	9.20 (5.62–15.05)	9.31 (5.65–15.35)	91 (0.5)	8 (4.5)	8.60 (4.35–17.00)	8.70 (4.35–17.39)	30 (0.9)	7 (7.3)	7.49 (3.73–15.04)	7.95 (3.95–16.00)	0.07
Acute respiratory distress syndrome	915 (4.3)	25 (9.2)	2.22 (1.48–3.33)	2.16 (1.44–3.23)	601 (3.4)	12 (6.8)	2.07 (1.16–3.71)	2.01 (1.13–3.59)	314 (9.1)	13 (13.5)	1.55 (0.87–2.75)	1.53 (0.87–2.70)	0.09
Mechanical ventilation	379 (1.8)	20 (7.3)	4.21 (2.70–6.57)	4.12 (2.62–6.48)	257 (1.4)	12 (6.8)	4.82 (2.72–8.55)	4.79 (2.67–8.61)	122 (3.5)	8 (8.3)	2.40 (1.19–4.84)	2.41 (1.17–4.95)	0.57
ICU admission	1,074 (5.0)	36 (13.2)	2.81 (1.99–3.97)	2.74 (1.93–3.89)	800 (4.5)	18 (10.2)	2.39 (1.48–3.87)	2.31 (1.42–3.76)	274 (7.9)	18 (18.8)	2.58 (1.57–4.25)	2.57 (1.54–4.28)	0.003

**Abbreviations:** HELLP = hemolysis, elevated liver enzymes, low platelet count; ICU = intensive care unit; RR = relative risk.

* Deliveries with discharge dates during March 2020–June 2021 were considered to occur during the pre-Delta period, whereas deliveries with discharges dates during July–September 2021 were considered to occur during the period of Delta predominance.

^†^ Models accounted for within-facility and within-woman correlation, and were adjusted for maternal age, race/ethnicity (Hispanic, non-Hispanic Black, non-Hispanic White, and non-Hispanic other), and primary payor (Medicaid, private insurance, and other).

^§^ Assessing for effect modification by period (pre-Delta versus period of Delta predominance), based on interaction term added to adjusted model.

^¶^ Includes chronic hypertension, gestational hypertension, chronic hypertension with superimposed preeclampsia, preeclampsia, HELLP syndrome, and eclampsia.

** Includes gestational hypertension, chronic hypertension with superimposed preeclampsia, preeclampsia, HELLP syndrome, and eclampsia.

^††^ Includes prepregnancy diabetes and gestational diabetes.

^§§^ Includes smoking (tobacco) complicating pregnancy, childbirth, or the puerperium.

^¶¶ ^Includes acute myocardial infarction, cardiomyopathy, heart failure/arrest during surgery or procedure, cardiac arrest/ventricular fibrillation, conversion of cardiac rhythm, incident ventricular tachycardia, ischemia, pulmonary edema/acute heart failure, and atrial fibrillation/atrial flutter/supraventricular tachycardia.

## Discussion

Although stillbirth was a rare outcome overall, a COVID-19 diagnosis documented during the delivery hospitalization was associated with an increased risk for stillbirth in the United States, with a stronger association during the period of Delta variant predominance. A previous study of pregnancies complicated by SARS-CoV-2 infection identified placental histopathologic abnormalities, suggesting that placental hypoperfusion and inflammation might occur with maternal COVID-19 infection (*5*); these findings might, in part, explain the association between COVID-19 and stillbirth. Among deliveries with COVID-19 documented during the delivery hospitalization, certain underlying medical conditions and markers of maternal morbidity, including the need for intensive care, were associated with stillbirth. Additional studies are warranted to investigate the role of maternal complications from COVID-19 on the risk for stillbirth. Further, given the differences observed before and during the period of Delta variant predominance, comparisons of placental findings might improve understanding of biologic reasons for the observed differences.

The rates of stillbirth in women without COVID-19 at delivery in this analysis (0.64% overall) were similar to the known prepandemic stillbirth rate of 0.59% (*6*). However, 0.98% of COVID-19–affected deliveries pre-Delta and 2.70% during the Delta period resulted in stillbirth. Data on the association between COVID-19 in pregnancy and stillbirth are emerging. Two metaanalyses found an association between COVID-19 during pregnancy and stillbirth but were unable to adjust for potential confounders (*2*,*4*). In a previous analysis of the PHD-SR data, comparing women with and without COVID-19 documented at the delivery hospitalization during March–September 2020, the risk for stillbirth was not significantly increased after adjusting for confounders (*3*). The current analysis includes an additional year of data, adding to the growing evidence that COVID-19 is associated with an increased risk for stillbirth.

Delta became the predominant variant of SARS-CoV-2 in the United States in July 2021.^§§§§^ The Delta variant is more infectious and is associated with increased risk for hospitalization compared with previous variants (*7*,*8*); however, nonpregnant patients are not more likely to have severe outcomes during hospitalization (*9*). In this analysis, the association between COVID-19 and stillbirth was stronger during the period of Delta predominance. Further studies that examine the effect of SARS-CoV-2 infection, including with the Delta variant, on fetal well-being are warranted.

The findings in this report are subject to at least seven limitations. First, the analysis relied on administrative data from hospital discharge ICD-10-CM codes; thus, identification of COVID-19 status, underlying medical conditions, gestational age, and stillbirths might be misclassified. Second, gestational age at SARS-CoV-2 infection was not available, and it is unknown whether COVID-19 diagnoses documented during the delivery hospitalization represented current or past infection. Third, many hospitals implemented universal SARS-CoV-2 testing among pregnant women assessed in labor and delivery units during spring 2020 (*10*), which would increase the detection of asymptomatic COVID-19. Laboratory information was unavailable for most hospitals in PHD-SR and therefore not used in this analysis; if participating hospitals had different screening practices, some patients with SARS-CoV-2 infection might have been missed or misclassified. In hospitals not conducting universal SARS-CoV-2 testing, women experiencing adverse outcomes during the delivery hospitalization, including stillbirth, might have been more likely to be tested for SARS-CoV-2 infection. Fourth, because outpatient records were not universally available, and linkage across different hospital systems was not possible, the analysis was restricted to codes included during the delivery hospitalization and did not examine COVID-19 diagnoses or underlying medical conditions recorded before the delivery hospitalization (i.e., during a prenatal visit). Fifth, whole genome sequencing data were not available to confirm the variant of SARS-CoV-2 for this analysis, and period was used as a proxy; however, the Delta variant accounted for >90% of U.S. COVID-19 cases during July–September 2021.^¶¶¶¶^ Sixth, it was not possible to assess vaccination status in this analysis. However, because COVID-19 vaccines are highly effective,***** and COVID-19 vaccination coverage among pregnant women was approximately 30% as of July 2021,^†††††^ most women with COVID-19 at delivery were likely unvaccinated. Finally, although the PHD-SR included a large population across U.S. Census divisions, it represents delivery hospitalizations from a convenience sample of reporting hospitals, limiting generalizability of results to the U.S. population.

This analysis adds to growing evidence of an association between COVID-19 in pregnancy and stillbirth, highlights that the risk for stillbirth associated with COVID-19 is affected by maternal morbidity, and demonstrates that the risk has increased during the Delta period. Further investigation from prospective studies is warranted to confirm these findings, identify the biologic mechanism for the observed increased risk for stillbirth with maternal COVID-19, and assess differences in risks relative to the timing and severity of infection and the contribution of maternal risk factors. In addition, further investigation of vaccine effectiveness during pregnancy, including prevention of stillbirth, is warranted. Most importantly, these findings underscore the importance of COVID-19 prevention strategies, including vaccination before or during pregnancy.

**Summary**

**What is already known about this topic?**

Pregnant women are at increased risk for severe disease from COVID-19, and COVID-19 is associated with an increased risk for adverse perinatal outcomes.

**What is added by this report?**

Among 1,249,634 delivery hospitalizations during March 2020–September 2021, U.S. women with COVID-19 were at increased risk for stillbirth compared with women without COVID-19 (adjusted relative risk [aRR] = 1.90; 95% CI = 1.69–2.15). The magnitude of association was higher during the period of SARS-CoV-2 B.1.617.2 (Delta) variant predominance than during the pre-Delta period.

**What are the implications for public health practice?**

Implementing evidence-based COVID-19 prevention strategies, including vaccination before or during pregnancy, is critical to reduce the impact of COVID-19 on stillbirths.

## Footnotes

*
https://covid.cdc.gov/covid-data-tracker/#datatracker-home

†
https://msdh.ms.gov/msdhsite/_static/23,23645,341.html

§ PHD-SR, formerly known as the PHD COVID-19 Database, is a large U.S. hospital-based, service-level, all-payor database that includes inpatient and hospital-based outpatient (e.g., emergency department or clinic) health care encounters from >900 geographically diverse nonprofit, nongovernmental, community, and teaching hospitals and health systems from rural and urban areas. PHD-SR represents approximately 20% of inpatient admissions in the United States. Data for this study represent a subset of 736 hospitals with delivery hospitalizations that contributed inpatient encounters to the PHD-SR during March 2020–September 2021. Updated PHD-SR data are released every 2 weeks; release date November 9, 2021, access date November 12, 2021. https://offers.premierinc.com/rs/381-NBB-525/images/PHD_COVID-19_White_Paper.pdf

¶ ICD-10-CM diagnostic and procedure codes pertaining to obstetric delivery: Z37.0, Z37.1, Z37.2, Z37.3, Z37.4, Z37.50, Z37.51, Z37.52, Z37.53, Z37.54, Z37.59, Z37.60, Z37.61, Z37.62, Z37.63, Z37.64, Z37.69, Z37.7, Z37.9, O75.82, O80, O82, 10D00Z0, 10D00Z1, 10D00Z2, 10D07Z3, 10D07Z4, 10D07Z5, 10D07Z6, 10D07Z7, 10D07Z8, 10E0XZZ; Diagnosis-related group delivery codes: 765, 766, 767, 768, 774, 775, 783, 784, 785, 786, 787, 788, 796, 797, 798, 805, 806, 807; Excluded codes for ectopic or molar pregnancies and pregnancies with abortive outcomes: O00, O01, O02, O03, O04, O07, O08, Z33.2, 10A0. Deliveries with the O82 code were excluded if they did not cooccur with another delivery code. Females aged 12–55 years were included. Multiple delivery events per woman during March 2020–September 2021 were included if the deliveries were >6 months apart.

** ICD-10-CM maternal diagnostic codes indicating a stillbirth: Z37.1, Z37.3, Z37.4, Z37.60, Z37.61, Z37.62, Z37.63, Z37.64, Z37.69, Z37.7. In multiple-gestation pregnancies, if a woman experienced multiple stillbirths, she was counted once as experiencing a stillbirth. If she experienced both a live birth and a stillbirth during one delivery hospitalization, she was also counted once as experiencing a stillbirth.

†† COVID-19 was identified using ICD-10-CM code U07.1 (COVID-19, virus identified) during April 2020–September 2021 or B97.29 (Other coronavirus as the cause of disease classified elsewhere) during March–April 2020.

§§ Includes smoking (tobacco) complicating pregnancy, childbirth, or the puerperium.

¶¶ Includes prepregnancy diabetes and gestational diabetes.

*** Includes chronic hypertension; gestational hypertension; chronic hypertension with superimposed preeclampsia; preeclampsia; hemolysis, elevated liver enzymes, low platelet count (HELLP) syndrome; and eclampsia

††† Includes acute myocardial infarction, cardiomyopathy, heart failure/arrest during surgery or procedure, cardiac arrest/ventricular fibrillation, conversion of cardiac rhythm, incident ventricular tachycardia, ischemia, pulmonary edema/acute heart failure, and atrial fibrillation/atrial flutter/supraventricular tachycardia.

§§§ Models accounted for within-facility and within-woman correlation, and were adjusted for maternal age, race/ethnicity (Hispanic, non-Hispanic Black, non-Hispanic White, and non-Hispanic other), primary payor (Medicaid, private insurance, and other), obesity, smoking, any diabetes, any hypertension, and multiple-gestation pregnancy.

¶¶¶ Models accounted for within-facility and within-woman correlation, and were adjusted for maternal age, race/ethnicity (Hispanic, non-Hispanic Black, non-Hispanic White, and non-Hispanic other), and primary payor (Medicaid, private insurance, and other),

**** 45 C.F.R. part 46, 21 C.F.R. part 56; 42 U.S.C. Sect. 241(d); 5 U.S.C. Sect. 552a; 44 U.S.C. Sect. Sect. 3501 et seq.
